# New formula of the green synthesised Au@Ag core@shell nanoparticles using propolis extract presented high antibacterial and anticancer activity

**DOI:** 10.1186/s13568-022-01450-6

**Published:** 2022-08-20

**Authors:** Nouran Rezk, Abdallah S. Abdelsattar, Salsabil Makky, Assmaa H. Hussein, Azza G. Kamel, Ayman El-Shibiny

**Affiliations:** 1 Center for Microbiology and Phage Therapy, Zewail City of Science and Technology, Giza, 12578 Egypt; 2 Center for X-Ray and Determination of Structure of Matter, Zewail City of Science and Technology, Giza, 12578 Egypt; 3 Faculty of Environmental Agricultural Sciences, Arish University, Arish, 45511 Egypt

**Keywords:** Silver nanoparticles, Gold nanoparticles, Multi-drug resistant, Nanocomposites, Antibacterial, Anticancer

## Abstract

Antimicrobial alternatives such as nanoparticles are critically required to tackle bacterial infections, especially with the emerging threat of antibiotic resistance. Therefore, this study aimed to biosynthesize Au–Ag nanoparticles using propolis as a natural reducing agent and investigate their antibacterial activity against antibiotic-resistant *Staphylococcus sciuri *(*S. sciuri*), *Pseudomonas aeruginosa *(*P. aeruginosa*), and *Salmonella enterica* Typhimurium (*S. enterica*), besides demonstrating their anticancer activity in cancer cell lines. The biosynthesized Au@AgNPs were characterized using UV–Vis spectrophotometer, Transmission Electron Microscopy (TEM), Zeta potential, Dynamic Light Scattering (DLS), Fourier Transformation Infrared (FTIR), and Scanning Electron Microscopy (SEM). Moreover, the detection of antibacterial activity was assessed through disc diffusion, the Minimum Inhibitory Concentration (MIC) and Minimum Bactericidal Concentration (MBC), time-killing curve, and detection of cell membrane integrity via SEM. As a result, the UV–Vis spectrum revealed the formation of Au@AgNPs in a single peak between 533 and 555 nm. Furthermore, FTIR analysis confirmed nanoparticles’ green synthesis due to the presence of carbon functional groups. The formulated Au@AgNPs showed antibacterial activity against both Gram-positive and Gram-negative bacteria. The MIC and the MBC of *P. aeruginosa* and *S. sciuri* were 31.25 µg/mL. However, nanoparticles were more effective on *S. enterica* with MIC of 7.5 µg/mL and MBC of 15.6 µg/mL. Furthermore, the time-killing curve of the three model bacteria with the treatment was effective at 50 µg/mL. Besides, SEM of the tested bacteria indicated unintegrated bacterial cell membranes and damage caused by Au@AgNPs. Regarding the anticancer activity, the results indicated that the biosynthesized Au@AgNPs have a cytotoxic effect on HEPG2 cell lines. In conclusion, this research revealed that the green synthesized Au@AgNPs could be effective antibacterial agents against *S. sciuri*, *P. aeruginosa*, and *S. enterica* and anticancer agents against HEPG2.

## Introduction

Antibiotic resistance in bacteria has emerged as a significant public health concern worldwide. In 2019, it was estimated that around five million deaths were caused by infections with antibiotic-resistant bacteria (Murray et al. 2022). The threat of antimicrobial resistance has emerged due to the overuse and misuse of commercial antibiotics and the lack of new antibiotics with new modes of action (Ventola 2015). Moreover, the rapid spread of antibiotic resistance can be due to the transfer of the resistance genes from one bacteria to another bacterial species (Lerminiaux and Cameron 2019; Sun et al. 2019). Developing new antibiotics requires a substantial financial investment and an extended period, which might be the least practical approach.

Therefore, new alternatives with higher potential and faster development time are urgently needed to combat antibiotic-resistant bacterial infections. Nanoparticles (NPs) have recently gained global attention as potential alternative antibacterial drugs. Nanoparticles have shown promising antibacterial therapy due to their unique physical and chemical properties. Similar to chemical antibiotics, nanoparticles have been widely studied against clinical infections. They exhibited targeted delivery to the infection site, good solubility profile, and long-term release with minimal side effects (Lee et al. 2019; Mba and Nweze 2021).

Nanoparticles can be either organic, such as liposomes, polymeric and micelles, or inorganic, such as metal nanoparticles. Both nanoparticles have been investigated to treat multiple diseases (Anselmo and Mitragotri 2016). In addition, the metallic nanoparticles showed promising results as antibacterial drugs against several multi-drug resistant pathogens, especially silver and gold nanoparticles. Silver nanoparticles (AgNPs) are the most widely used since they have effective antibacterial activity, low cost, low cytotoxicity and immunological response (Yin et al. 2020).

Furthermore, gold nanoparticles (AuNPs) exhibited good antibacterial activity due to their stability, non-toxicity, biocompatibility, and large specific surface area for binding with target bacteria, besides the significant antibacterial activity against Gram-positive and negative bacteria (Gu et al. 2021). Gold-silver bimetallic nanoparticles (Au-AgNPs) have demonstrated a synergistic activity, generating bifunctional effects compared to their monometallic counterparts. The primary benefits of Au@AgNPs are biocompatibility, reduced silver nanoparticles (AgNPs) toxicity toward healthy human cells and significant antibacterial activity (Deng et al. 2021; Villalobos-Noriega et al. 2021; Rabiee et al. 2022).

The traditional physical and chemical strategies for metallic nanoparticle synthesis have exhibited severe side effects by using hazardous agents that lead to toxicity, carcinogenicity, and environmental pollution. Moreover, they hinder the use of nanoparticles in biomedical applications (Jamkhande et al. 2019). For instance, the chemical approach of nanoparticle synthesis uses silver solutions, reacts with a proper reducing agent to produce metallic nanoparticles, and capping agents are used for stabilization. However, they employ hazardous and highly toxic compounds, including organic solvents, reducing agents, and stabilizers leading to environmental pollution (Gupta and Xie 2018). In addition, they use very expensive capping and reducing agents (Javed et al. 2020). Moreover, physical methods are inefficient and require a huge amount of energy to maintain proper pressure and temperature during the whole process of synthesis (Iravani et al. 2014; Pourzahedi and Eckelman 2015; Caramazana et al. 2018; Ijaz et al. 2020).Therefore, the development of metallic nanoparticles using green synthesis has attracted significant attention (Mukherjee et al. 2014; Andal et al. 2018; Zhang et al. 2020; Deng et al. 2021).

Green synthesis is an approach to nanoparticle synthesis using biological agents, including fungi (Honary et al. 2013), algae (Chugh et al. 2021), bacteria (Truong et al. 2022), and plant extracts (Varadharaj et al. 2020). It employs biological and eco-friendly substances, such as specific enzymes, amino acid groups, proteins, sugars, vitamins, and biodegradable polymers to be reducing agents, end-capping agents and solvents. As a result, less energy will be consumed, and no toxic or hazardous reagents will be required (Hussain et al. 2016; Alsammarraie et al. 2018; Huston et al. 2021). Therefore, it has many advantages over chemical and physical methods, such as being environmentally friendly, cost-effective, biocompatible, easily scaled up for mass production and safe for human cells and animals (Honary et al. 2015; Barabadi et al. 2020; Virmani et al. 2020; Ying et al. 2022).

One of the most commonly used natural reducing and stabilizing agents is propolis. Propolis is a natural product collected by bees from various plant sources with pharmacological activities such as anticancer, antioxidant, anti-inflammatory, and antimicrobial effects (Ong et al. 2019). In addition, some research studies have used propolis as a reducing agent to biosynthesize gold nanoparticles due to its high concentration of flavonoids, polyphenolic acids, terpenoids, and other molecules that can reduce Au^+3^ to Au (Roy et al. 2010; Righi et al. 2011; Gatea et al. 2015).

Hence, this study aimed to investigate the characterization of green synthesized Au@AgNPs with a new formula using propolis as a natural reducing agent with a simple, eco-friendly, low-cost method, in addition to demonstrating their antibacterial activity against Gram-positive antibiotic-resistant *S. sciuri*, Gram-negative *P. aeruginosa*, and *S. enterica* and the anticancer activity against hepatocellular carcinoma cell lines.

## Materials and methods

### Preparation of propolis extracts

The propolis was prepared with modifications by the ethanol extraction method (Bankova et al. 2021). Briefly, 0.4 g of the raw propolis was added to 10 mL of 80% ethanol for 2 h at 60 °C, followed by incubation for another 2 h at 80 °C. Then, the extraction was centrifuged at 4000×*g* for 15 min. Finally, the supernatant extract was drawn and filtered with a 0.45 μm syringe filter to ensure its purification.

### Green synthesis process for the core and shell Au@AgNPs

The core–shell production of Au@AgNPs was obtained by adding 1 mM of HAuCl_4_ into deionized water containing 3% ethanolic propolis extracts. The mixture was left on a hot plate at 85 °C for 120 min with contentious stirring at 400 rpm. Then, the formulated core, AuNPs, was used as a reducing agent for the shell, Au@AgNPs; 2 mM AgNO_3_ was added for another 90 min in the same condition of forming the AuNPs. The colors in each step were monitored as an indicator for transforming an ion into a nanoparticle form.

### Characterization of AgNPs

The UV–visible spectrophotometer (Jenway 7200, Staffordshire, UK) was employed to determine the biosynthesized Au@AgNPs*.* One (1)  mL of the ten-fold diluted Au@AgNPs was placed in a spectrophotometer with an applied wavelength range of 340–800 nm. For nanoparticle imaging, TEM (1230 JEOL Tokyo, Japan) was used to investigate the size and shape of core–shell Au@AgNPs via dropping the nanoparticles on the surface of carbon coating Cu-grids before scanning the sample with TEM. In addition, the overall charge of the formulated core–shell Au@AgNPs was measured via Zetasizer Nano ZS (Malvern, UK). The Zeta-potential measurements were conducted in a disposable cell at room temperature from − 100 to 100 mV and analyzed using Zetasizer software. In addition, the size distribution was analyzed using dynamic light scattering (DLS). Moreover, the functional groups in the biosynthesized Au@AgNPs were predicted by FTIR. The FTIR analysis was conducted by the (Agilent system Cary 360 FTIR) model, which ranges from 4000 to 400 cm^−1^. Furthermore, the morphological analysis of AuNPs and the green synthesized Au@AgNPs was conducted using the SEM (JEOL, JSM-IT 200, Japan). The figures from SEM were obtained after gold coating by an Ion sputter evaporator (JEOL, JFC-1100E, Japan). Finally, the elements’ detection was predicted using the same microscope equipped with the energy dispersive X-ray analysis (EDX).

### Antibacterial activity

#### Bacterial culture

Three different bacterial types were used in this study; *S. sciuri* MW41588 and *P. aeruginosa* OL375153 (obtained from the library of the center for microbiology and phage therapy, Zewail City, Egypt), in addition to *S. enterica* NCTC 13348. The bacterial strains were streaked to be refreshed on selective growth media agar of Mannitol salt agar for *S. sciuri*, Cetrimide agar for *P. aeruginosa*, and MacConkey agar for *S. enterica* at 37 °C for 18–24 h.

### Zone of inhibition assay

The antibacterial efficacy of the core–shell Au@AgNPs was studied using the disc diffusion assay. First, using sterile cotton swabs, overnight bacterial cultures were swabbed uniformly on Tryptone Soya Agar (TSA) plates. Next, filter paper discs (of 6 mm in diameter) were autoclaved and loaded on TSA plates. After that, 10 µL of Au@AgNPs were added to each disc. In addition, an empty disc was used as a negative control. Then, the plates were incubated upside-down at 37 °C for 24 h. Finally, the inhibition zones were measured for each disc (Bauer et al. 1966).

### MIC and MBC

The MIC assay was conducted on 96-well flat-bottom plate using the standard broth microdilution method (Parvekar et al. 2020). MBC test was performed by withdrawing the liquid media from each well of 96-well plates into the TSA plate. The spots with no growth at the lowest concentration are considered the MBC (Loo et al. 2018). First, 100 µL of bacterial culture (10^7^ CFU/mL) from each strain were added to 100 µL of Au@AgNPs (500 µg/mL). Then, the nanoparticles’ concentration was diluted by half to obtain the following concentrations: 1.8, 3.7, 7.5, 15.6, 31.2, 62.5, 125, and 250 μg/mL. Next, the microliter plate was incubated at 37 °C overnight. The MIC values were measured as the lowest value of Au@AgNPs that can inhibit the growth of bacteria. Furthermore, The MBC was calculated as the lowest concentration that killed %99.99 of the bacteria.

### Growth kinetics assay

The antibacterial activity of different concentrations of Au@AgNPs during a short period was investigated via a time-killing curve. Briefly, 100 µL of bacterial culture with ~ 0.15 OD_600_ was incubated with Au@AgNPs (0.05, 0.16, 0.5, 1.6, 5, 16.6, 50 µg/mL) in a sterile 96-well plate. Untreated bacterial culture was used as a control. The plate was incubated at 37 °C for 315 min (~ 5 h) with a continuous absorbance reading at OD_600_ every 15 min using FLUOstar Omega Microplate Reader.

### Cell membrane integrity

The bacterial cultures were grown at 37 °C to reach OD_600_ of 0.1. Next, *P. aeruginosa was* treated with Au@AgNPs (0.5 μg/mL). Additionally, *S. enterica* and *S. sciuri* were incubated with 5 μg/mL Au@AgNPs as a final concentration for 7 h. Then, the cells were centrifuged at 7000 rpm for 10 min at 20 °C before fixation. Finally, the SEM was used to image the surface of different bacterial cells after coating them with gold.

### Cytotoxicity assay

HEPG2 (hepatoblastoma) cell lines were seeded in a sterile 96-well plate with a seeding density of 5 × 10^3^ cells/well in Dulbecco Modified Eagle’s Medium (DMEM) culture media supplemented with 10% fetal bovine serum (FBS), 100 mg/mL of streptomycin, and 100 units/mL of penicillin as an antibacterial and antifungal agent. The plate was incubated for 24 h at 37 °C and 5% CO_2_. After incubation, powder Au@AgNPs were suspended into DMEM and added to the seeded cells with various concentrations as follows: 10, 20, 50, 100, 200, 500 and 1000 µg/mL in compassion to the control (without Au@AgNPs), then incubated for 72 h at 37 °C and 5% CO_2_. Then, all media were discarded carefully, then 90 µL of DMEM media and 10 µL of MTT labeling reagent (final concentration 0.5 mg/mL) was added to each well, and the microplate was incubated for 4 h at 37 °C and 5% CO_2_. The media was then removed carefully, and 150 µL of DMSO was added to each well to allow solubilization of the formazan crystals and the plate was incubated for 20 min. After incubation, the absorbance at 570 nm was measured using FLUOstar Omega Microplate Reader. Experimental concentrations and control were performed on technical triplicates and two biological replicates.

## Results

### The characterization of Au@AgNPs through color change during the preparation

**Fig. 1:**
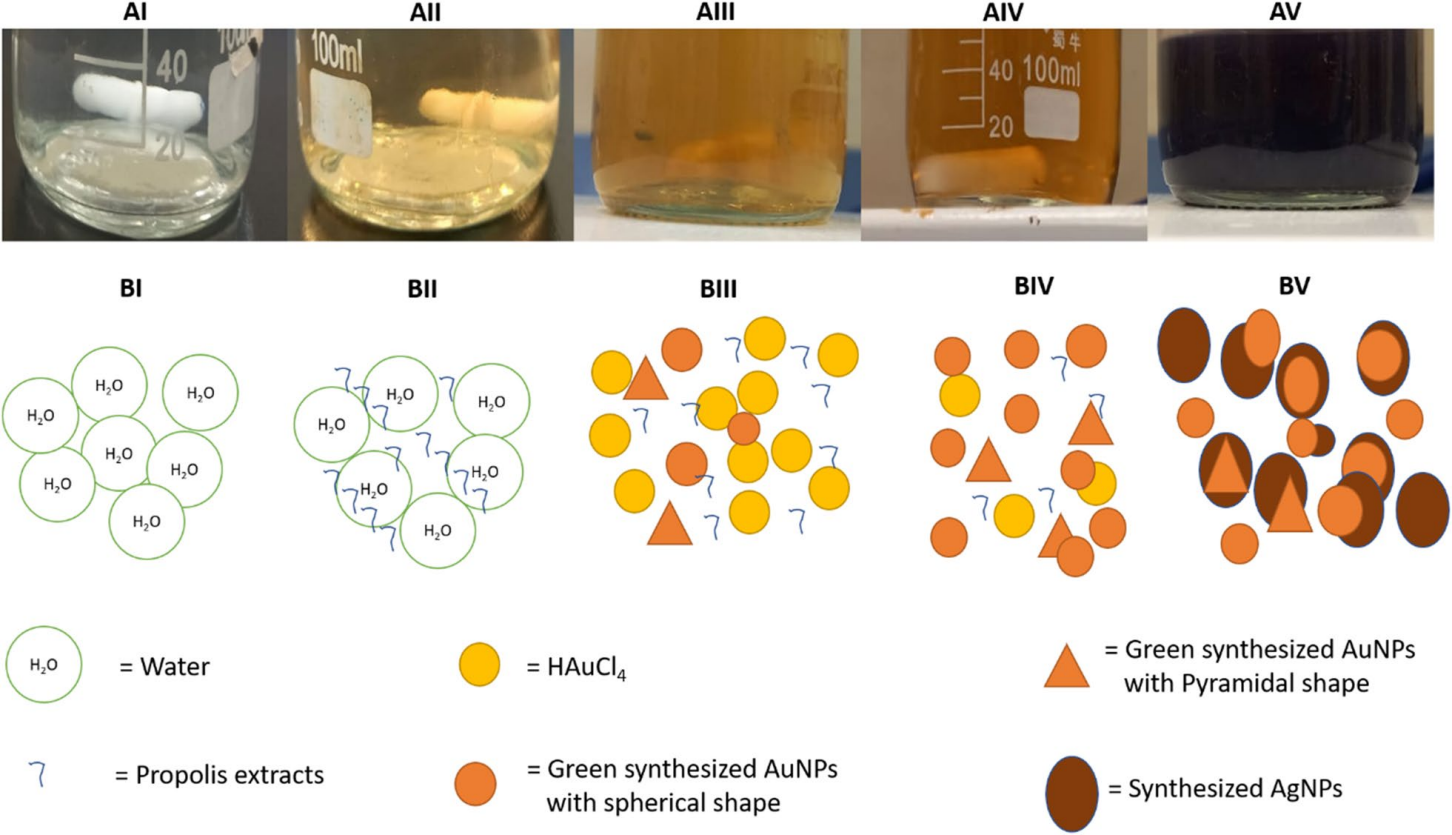
Successive color changes during the synthesis of Au@AgNPs using ethanoic extract of propolis. **AI** and **BI**. The water is at zero point. **AII** and **BII** The water containing 3% propolis extract at zero point. **AIII** and **BIII** An intermediate step in forming the AuNPs after 80 min. **AIV** and **BIV** A late step in synthesizing the AuNPs after 120 min. **AV** and **BV** A final step in producing the Au@AgNPs, after 90 min of adding AgNO_3_ to green synthesized AuNPs

The formation of Au@AgNPs capping with propolis extract was confirmed by changing the color during the multi-step preparation. First, the colorless water changed to yellowish after adding the propolis extract (Fig. 1AI and AII). Then, it is changed to brown, confirming the fast reduction of Au^+^ to Au^0^ in the solution (Fig. 1AIII and AIV). Finally, the color became purple after adding the AgNO_3_ in the presence of heat and light as inducers (Fig. 1AV).

### The characterization of Au@AgNPs using UV–vis spectrum, FTIR, and zeta potential and DLS

**Fig. 2:**
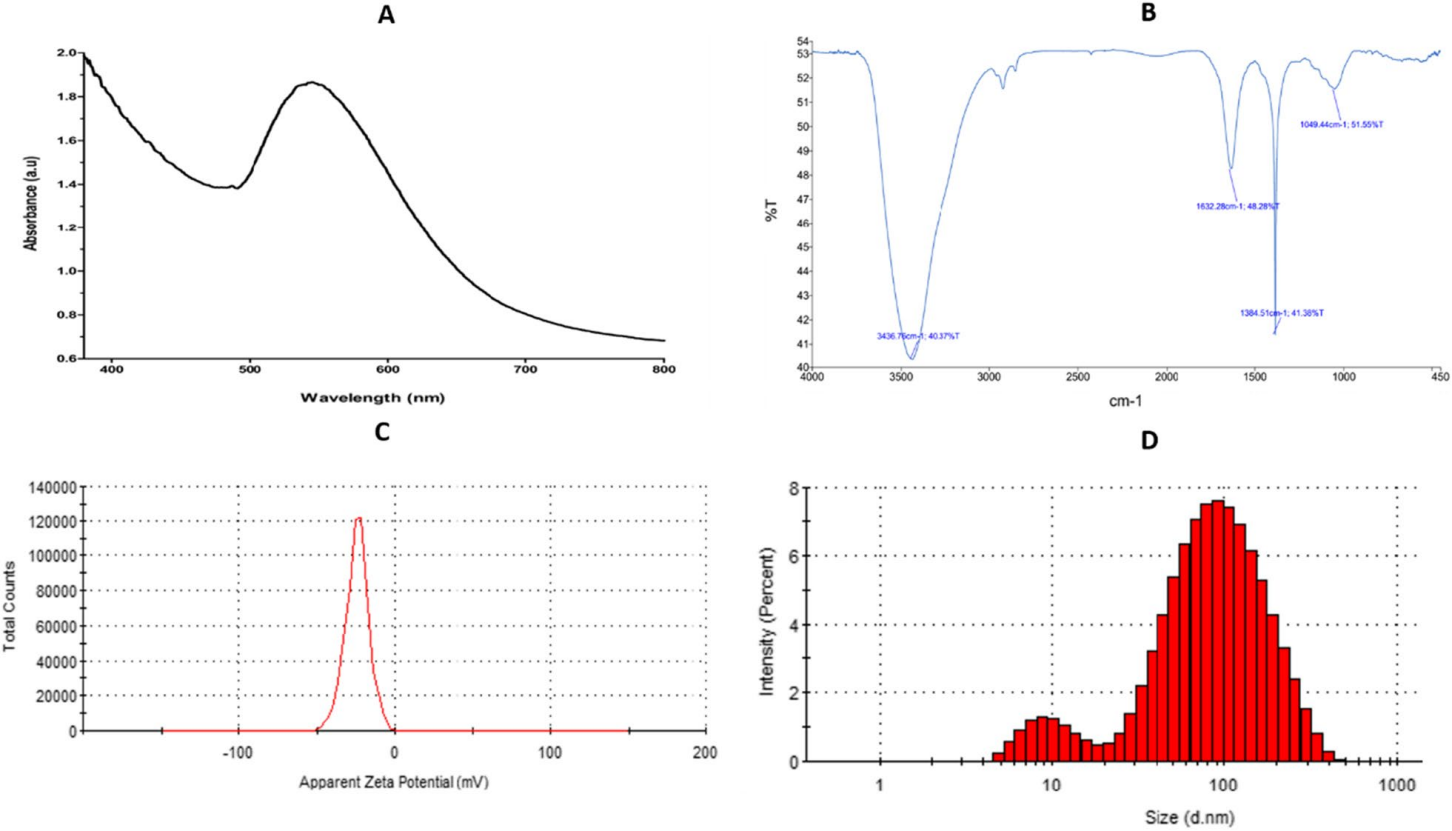
Various characterization techniques for Au@AgNPs. **A** UV–vis spectrum, **B** FTIR, **C** the zeta-potential diagram, and **D** DLS diagram

The formulation of Au@AgNPs was analyzed using UV–visible spectrophotometers (Fig. 2A). The UV–Vis spectrum resulted in a peak with maximum absorption between 533 and 555 nm. In addition, the FTIR analysis showed various peaks, as shown in Fig. 2B, including 3436 cm^−1^, 1632 cm^−1^, and 1049 cm^−1^. In addition, the zeta potential value was analyzed to determine the overall charge of the formulated nanoparticles; a peak at − 24 mV was displayed for Au@AgNPs (Fig. 2C). Finally, the results of DLS showed size distribution with a significant beak representing 85% of the sample, which a diameter equal to 108 ± 68 nm.

### The characterization of Au@AgNPs using EDX

**Fig. 3:**
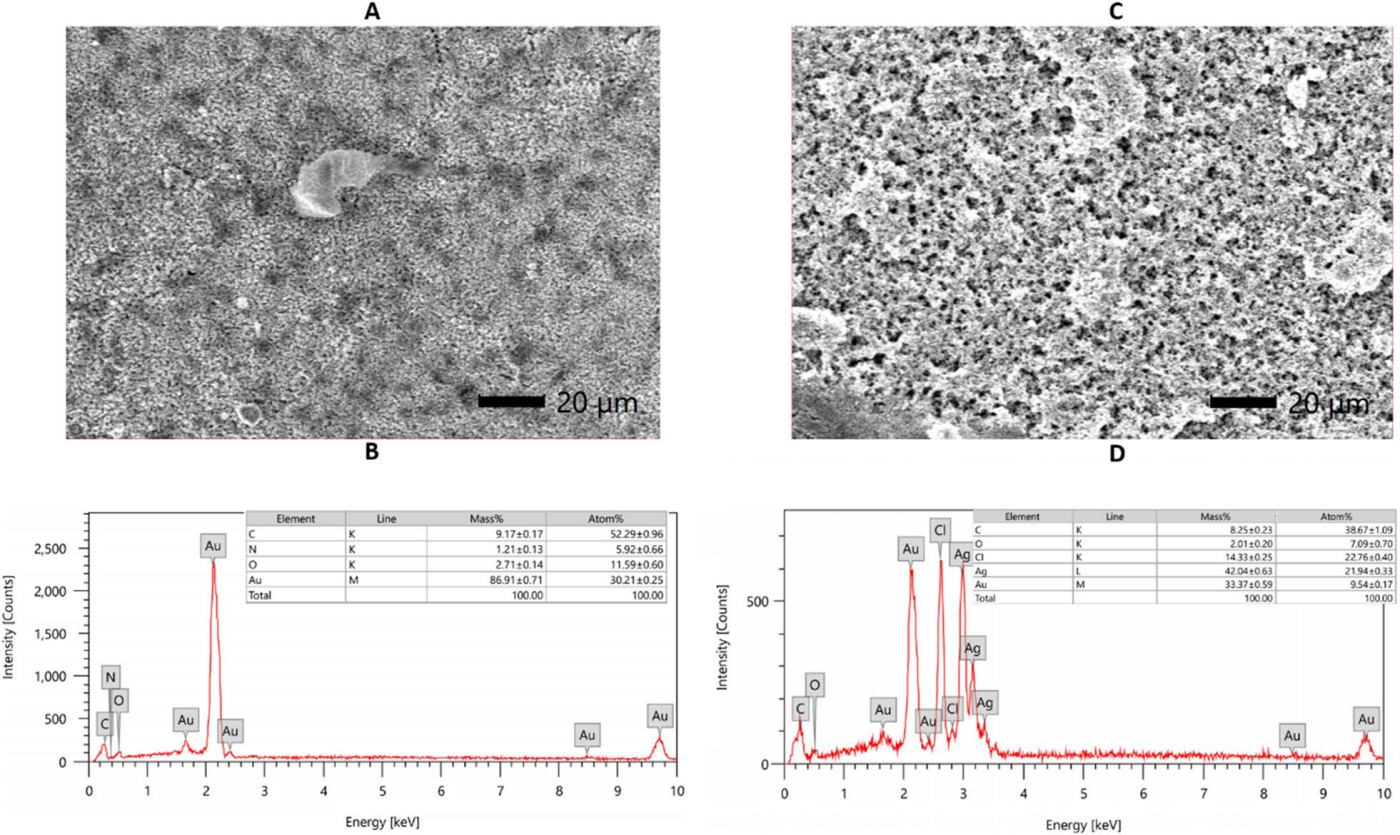
The EDX analysis of **A**, **B** AuNPs and **C**, **D** Au@AgNPs

The elemental analyses of AuNPs and Au@AgNPs were measured using the EDX. Figure 3A showed the field of AuNPs with the highest gold mass, 86.7%, then 9.1% carbon, followed by traces of oxygen and nitrogen (Fig. 3B). On another side, Fig. 3C and D illustrated the field of Au@AgNPs with the highest value of silver at 42%, gold 33.3%, then chlorine with 14.3%, around 8.3% carbon and traces of oxygen.

### The characterization of Au@AgNPs using SEM and TEM

**Fig. 4:**
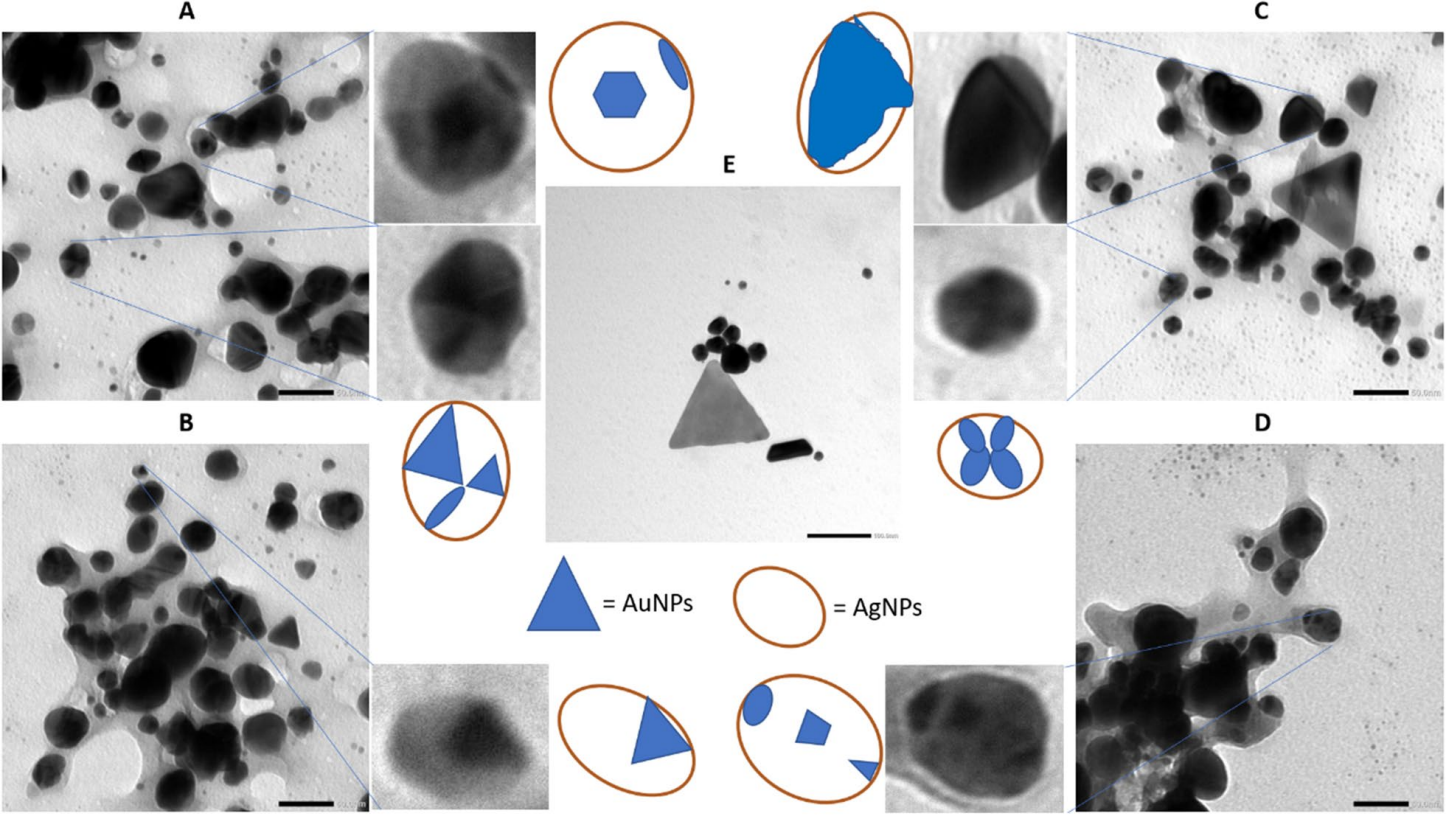
The TEM images and suggested structures for **A**–**D** Au@AgNPs and different fields, with a scale bar of 50 nm, and **E** for AuNPs, with a scale bar of 100 nm

**Fig. 5:**
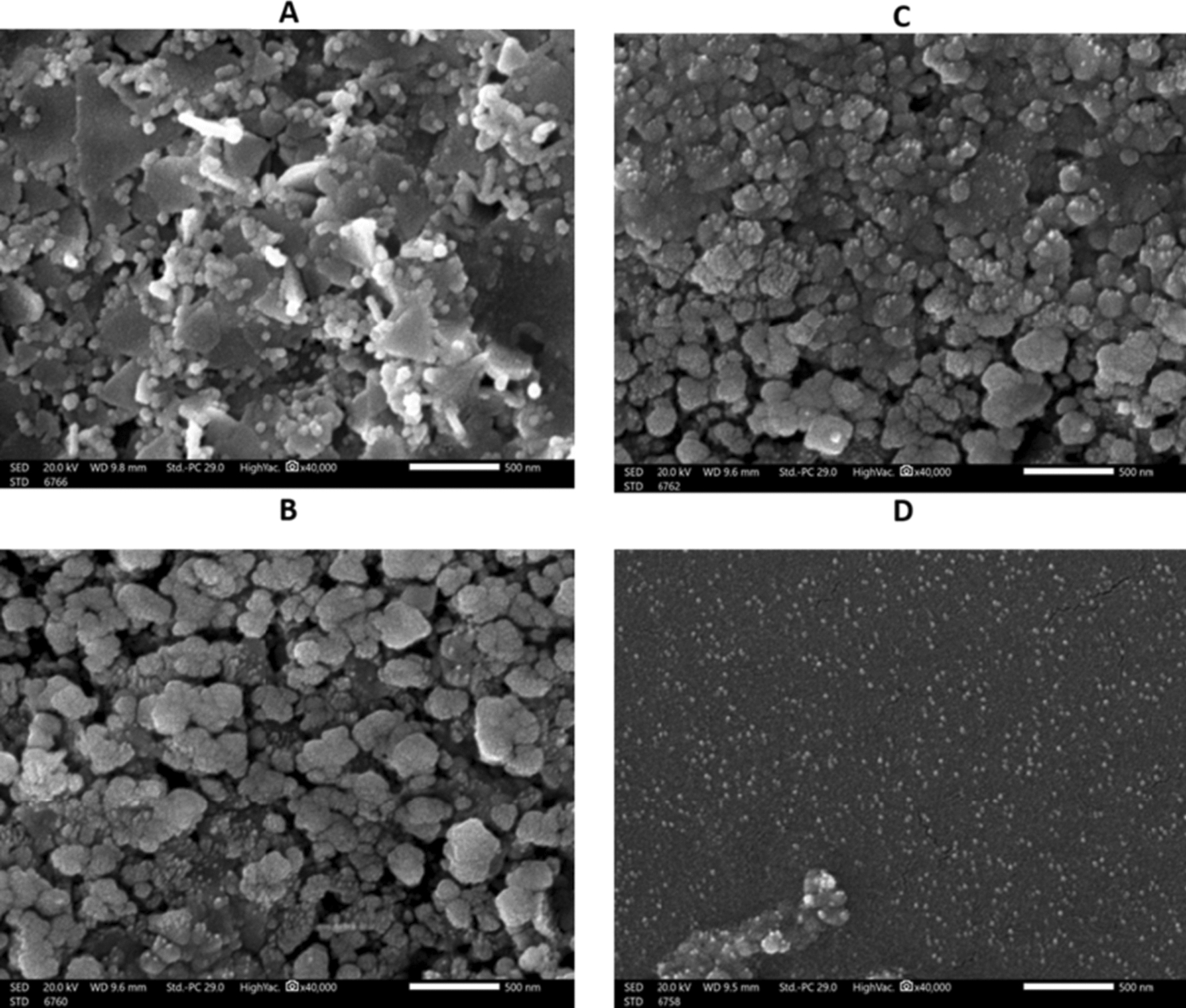
The SEM images for **A** AuNPs and **B**–**D** for Au@AgNPs in different fields

For further confirmation of the formulation of Au@AgNPs in the form of a core@shell structure, the nanocomposite sample was tested using SEM and TEM (Figs. 4 and 5). For AuNPs, the SEM and TEM micrographs in Figs. 4E and 5A displayed three different morphologies: triangles, spheres, and trapezoids with sizes less than 50 nm. However, Au@AgNPs, illustrated in Figs. 4A–D and  5B–D, appeared as spheres containing two degrees of color: the darker one refers to the gold, and the faint color is the silver.

### The antibacterial activity of the Au@AgNPs

**Fig. 6:**
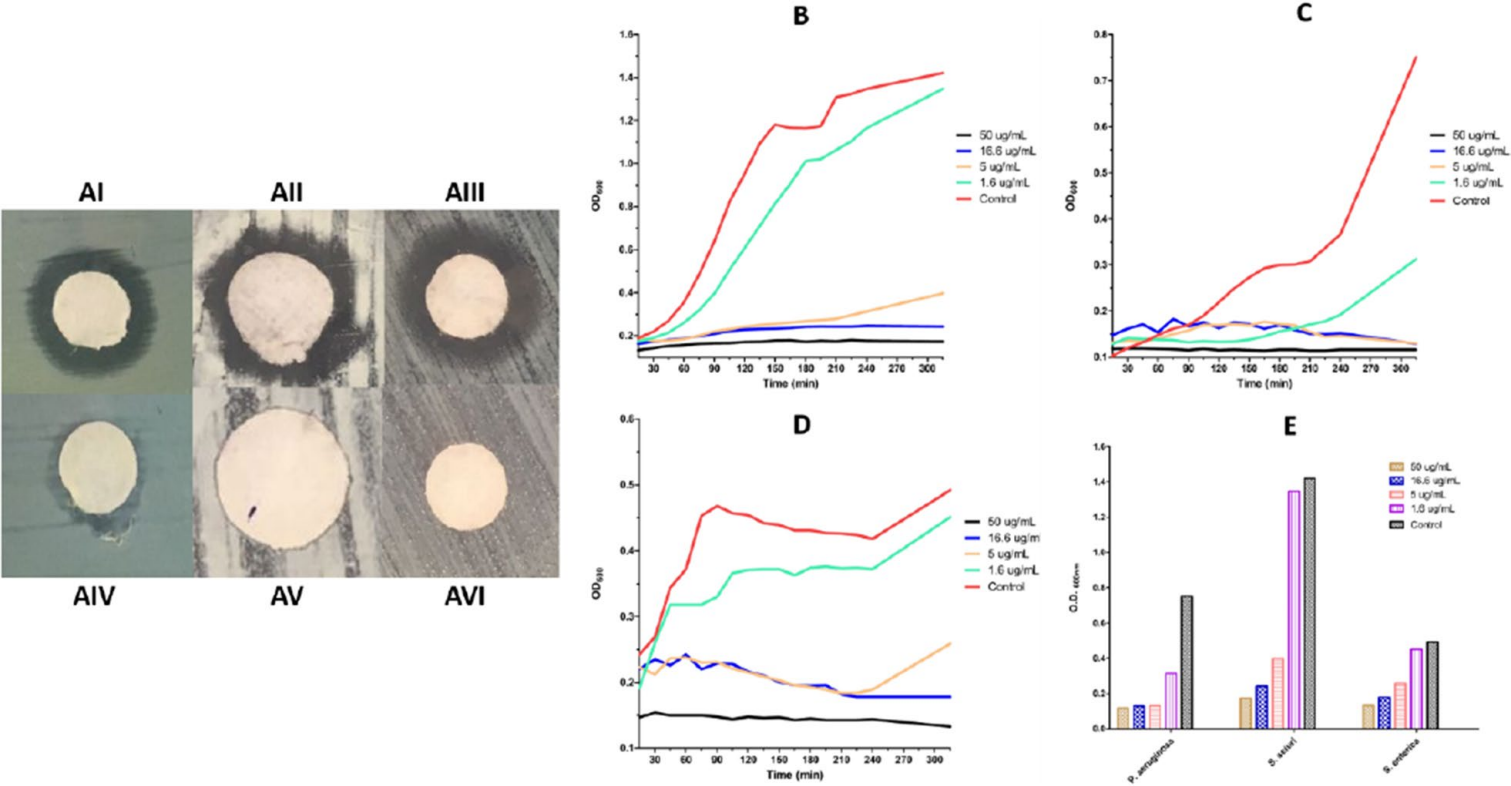
The antibacterial activity of Ag-AuNPs against the bacteria **A** by disc diffusion method against **AI** *P. aeruginosa* **AII** *S. sciuri* **AIII** *S. enterica* using Ag-AuNPs and the negative control for the same bacteria (**AIV**, **AV**, and **AVI**) respectively. Moreover, the time-killing curve during 315 min of **B** *S. sciuri* **C** *P. aeruginosa* **D** *S. enterica* after being treated with different concentrations of Ag-AuNPs. The chart **E** represents the readings at OD_600_ after 315 min

As illustrated in the results, the formulated Au@AgNPs have antibacterial activity against Gram-positive and Gram-negative bacterial strains. Nonetheless, the Au@AgNPs displayed variable antibacterial effects on the three tested bacteria. For instance, the MIC and the MBC for *P. aeruginosa* and *S. sciuri* were 31.25 µg/mL. Au@AgNPs were effective antibacterial agents against *S. enterica* with MIC of 7.5 µg/mL and MBC of 15.6 µg/mL. Furthermore, the disc diffusion test confirmed the antibacterial activity of the formulated Au@AgNPs on TSB plates overlaid with each of the three model bacteria as in Fig. 6A–AVI. Discs of 10 µL of Au@AgNPs were added to each plate and ddH_2_O as a negative control. The results showed that Au@AgNPs could inhibit the growth of the cultured bacteria, with inhibition zones close to 8 mm. In addition, the time-killing curve of the three model bacteria responded to the Au@AgNPs treatment in a concentration-dependent manner, where Au@AgNPs (50 µg/mL) was effective against all bacteria. However, as the concentration of the used Au@AgNPs decreased, their antibacterial activity decreased, as in Fig. 6B–E. The present graphs of Au@AgNPs (1.6 µg/mL) displayed the minimum antibacterial activity that was close to the untreated bacteria. Whereas Au@AgNPs (5 µg/mL) showed better activity against the three bacteria at the first three hours and a half (~ 210 min), then *S. sciuri* and *S. enterica* started developing a secondary bacterial growth. However, in 16.6 and 50 µg/mL concentrations, the antibacterial activity lasted more than five hours (350 min).

**Fig. 7:**
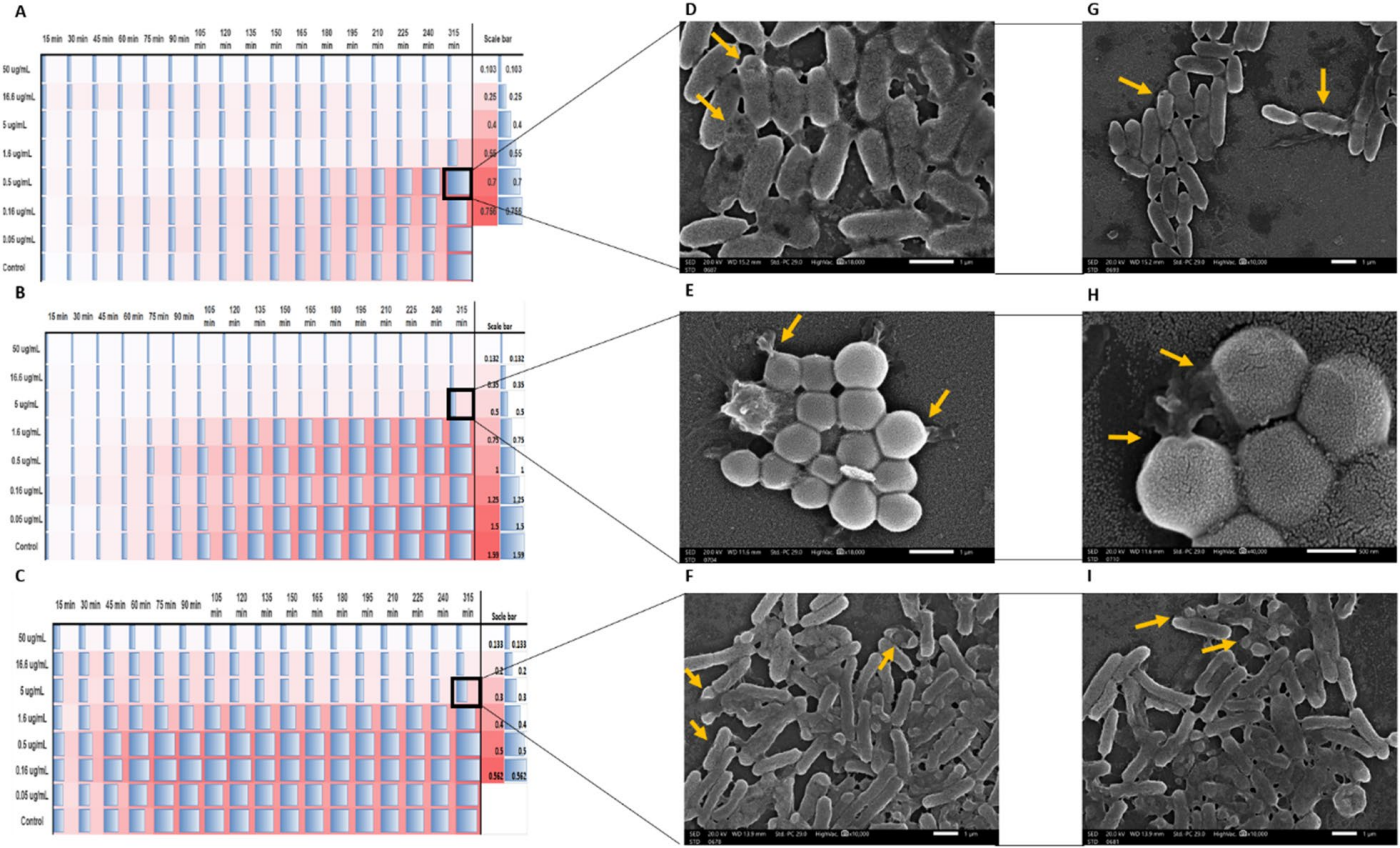
A heatmap and SEM images for the bacteria damage after adding the Au@AgNPs. The heat map of **A** *P. aeruginosa* **B** *S. sciuri* **C** *S. enterica*. The SEM images for the bacterial damage in the cell membrane of **D**, **G** *P. aeruginosa*, **E**, **H** *S. sciuri*, and **F**, **I** *S. enterica* after exposure to the Au@AgNPs in the concentrations marked in the black box (**A**–**C**). The orange arrows refer to the damage to the cell membrane

The dynamic interactions between the formulated Au@AgNPs and the three bacterial strains was studied, separately. The bacterial strains were incubated individually with different concentrations of the Au@AgNPs for five hours. The results displayed a continuous growth of the untreated bacteria, and the inhibition of the bacteria treated with Au@AgNPs was achieved in a concentration-dependent manner, as illustrated in the heatmaps (Fig. 7A–C). Moreover, the bacteria treated with a sub-MIC dose of Au@AgNPs were depicted using SEM to observe the damage caused by Au@AgNPs. The bacteria in SEM micrographs present unintegrated bacterial cell membranes (Fig. 7D–I).

### Cytotoxicity of Au@AgNPs

**Fig. 8:**
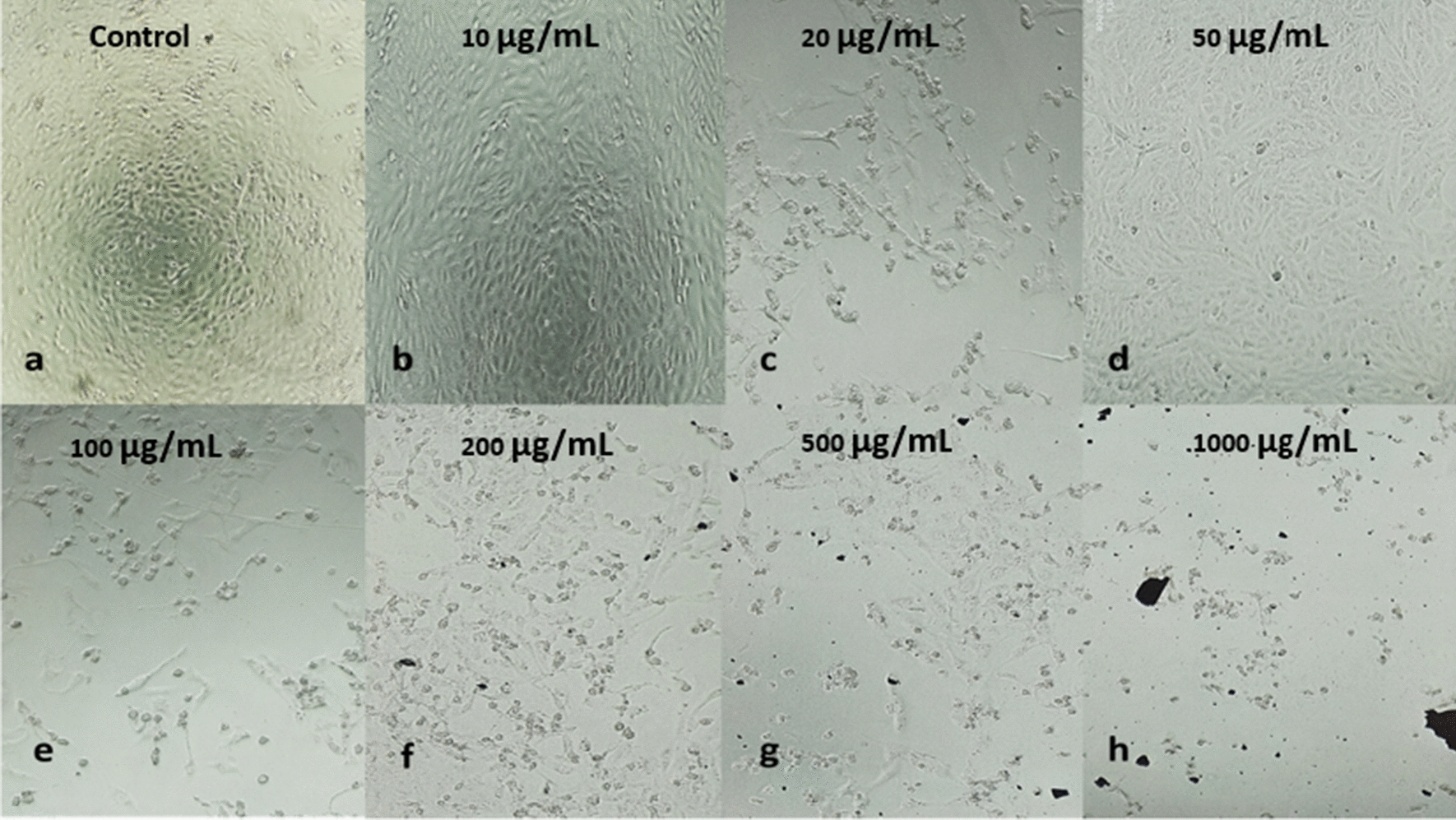
HEPG2 cell lines after 72 h incubation with various concentrations of Au@AgNPs compared with the control (without treatment)

**Fig. 9:**
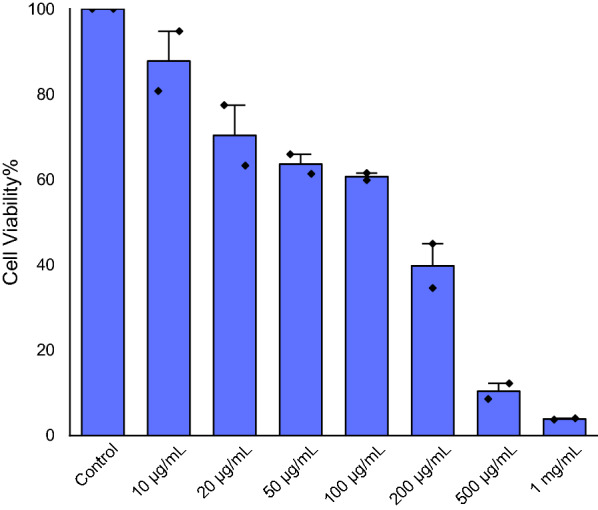
The cell viability percentage of HPEG2 after being treated with various concentrations of Au*@*AgNPs (10, 20, 50, 100, 200, 500 and 1000 µg/mL). The results represent the means of three replicates, with two biological replicates and the error bars represent the standard error of the mean

The cytotoxicity effect of Au@AgNPs was done on the HEPG2 cell line, and the cell viability was determined by MTT assay. The experiment was done for seven different concentrations (10, 20, 50, 100, 200, 500 and 1000 µg/mL). The results showed a reduction of the cell viability with morphological deterioration and loss of consistency with increasing the concentrations on Au@AgNPs (Fig. 8). The cytotoxicity assay displayed that by increasing the concentration from (10–1000 µg/mL), the cells were highly affected, with IC50 = 140 (Fig. 9). At low concentration (10 µg/mL), there was no significant decrease in the cell viability compared to the control (*P*-value = 0.45). However, when the concentration increased up to 1000 µg/mL, there was a significant reduction in the cell viability compared to the control. For instance, the cell viability after treatment with each concentration is as the following: 20 µg/mL = 88% (P-value = 0.01), 50 µg/mL = 70% (P-value = 0.003), 100 µg/mL = 64% (P-value = 0.002), 200 µg/mL = 40% (P-value = 0.0001), 500 µg/mL = 10% (P-value = 0.0001) and 1 mg/mL = 4% (P-value = 0.0001).

### Discussion

Following the spread of multidrug-resistant (MDR) bacteria, alternatives to antibiotics were pursued to face the antibiotic resistance challenge. Among the promising antimicrobial agents are biosynthesized nanoparticles. As the name implies, the biosynthesized nanoparticles depend on various biological agents to act as capping and reducing factors for the synthesized nanoparticles. In addition to the safety and biocompatibility of the biological reducing agents, they contain many phytochemicals that further enhance the properties of the formulated nanoparticles (Saravanan et al. 2021).

Accordingly, this study focused on the biosynthesis of Au@AgNPs with a new formula by using propolis as a natural reducing agent with a simple, eco-friendly, cheap method. Moreover, the biosynthesized Au@AgNPs were fully characterized using different techniques including UV–Vis spectrum, FTIR, Zeta potential, and EDX and visualized using TEM and SEM in addition to testing their antibacterial, anticancer and cytotoxicity actvities.

### The characterization of Au@AgNPs

The propolis (glue secreted by the honeybees) was used as a biological reducing agent. Propolis has high medical value since it has antimicrobial, antiviral activities, anticancer, antioxidant, and anti-inflammatory effects. That is why propolis was employed here as a biological reducing agent to the prepared nanoparticles.

To confirm the biosynthesis of AuNPs using propolis, the color of the HAuCl_4_ solution was monitored over time. When the color changes to brown, it indicated the nanoform formation. Then, AgNO_3_ was added to formulate the shell on the AuNPs core. The change of color into purple following around 90 min stated the construction of the AgNPs. The nanoparticle formation was then confirmed and characterized using various techniques: UV–Vis spectrum, FTIR, Zeta potential, and EDX and visualized using TEM and SEM.

The UV–Vis spectrum is usually used to confirm the biosynthesis of nanoparticles, which reveals the excitation of surface plasmon resonance (SPR). Here, SPR was perceptible in the peak produced by the nanoparticle between 533 and 555 nm, confirming the nanoform’s presence. Furthermore, the resulted data have a longer wavelength than the previously reported Au@AgNPs (Villalobos-Noriega et al. 2021), which can be explained by the high gold content in the sample (Loiseau et al. 2019). Thus, we are investigating the other varied properties due to the high concentration of HAuCl_4_ in the core@shell formula compared to the previously reported data.

FTIR analysis was done to investigate the surface functional groups of the formulated nanoparticles. It reveals four prominent peaks. One strong peak at 3436 cm^−1^ is for hydroxyl groups, then 1632 cm^−1^ is associated with stretching (C=C) and the band at 1049 cm^−1^ indicates the presence of C–O stretch (Zhou et al. 2015). However, the appearance of a sharp peak at 1384 cm^−1^ indicates this is due to the N=O bend. The presence of functional groups related to carbon confirms the successful green synthesis of nanomaterial from propolis. Moreover, the negative zeta-potential value (− 24.4 mV) reveals that the nanoparticles have high repulsive interactions which prevent the aggregation of the nanoparticles and provide stability of the metal colloids (Ajitha et al. 2015; Rezazadeh et al. 2020). In addition, the relatively high negative zeta-potential value might be due to the propolis extract’s polyphenolic molecules that attach to the nanocomposite surface (Babu et al. 2017).

To further confirm the FTIR results and quantify the ratio between the nanoparticles’ components before and after the shell formation with AgNPs, EDX analysis was done. The results showed that 90% of the overall mass of AuNPs formulated before the coating is for the gold atoms, followed by around 10% of carbon, oxygen and nitrogen atoms. After the coating with AgNPs, most of the mass of Au@AgNPs belongs to silver atoms, 42%, followed by gold atoms, 33.4%. A small peak appeared in Au@AgNPs analysis, which belongs to the chlorine from HAuCl_4;_ it is not found in AuNPs. The presence of a chlorine peak may reveal the role of chlorine in forming the nanocomposite of Au@AgNPs. Altogether, the EDX readout, besides the information from FTIR, suggests that the presence of elements such as carbon, nitrogen, and oxygen reveals the high interaction between propolis extract and the nanocomposites (Fafal et al. 2017). In addition, the purity of the formulated Au@AgNPs is higher than the previous data that showed contamination with copper (Wang et al. 2016).

SEM and TEM micrographs were analyzed to get a closer look at the morphology, size and layers of the formulated Au@AgNPs. The SEM micrograph of AuNPs displayed the multiple triangle structures characteristic of the AuNPs, whereas the SEM of Au@AgNPs presented minimal to no triangle structures. While the dominant structure for the AuNPs is the triangular shape, the majority of Au@AgNPs are spherical, as indicated before (Kuppusamy et al. 2017; Beldjilali et al. 2020; Herbin et al. 2022). This observation might agree with the hypothesis of forming a core and shell structure of Au@AgNPs. On the other hand, the SEM and TEM micrographs display the sizes of the formulated AuNPs and Au@AgNPs, ranging from 20 to 40 nm. It is expected that the formulated nanoparticles are in the size range that make them of high activity against their targeted bacteria (Zhang et al. 2018), as they have a large surface area that contributes to the high interaction with the cell membrane and organelles. Although the TEM images illustrate the formation of different structures, the EDX mapping is still needed to confirm the suggested structures (Lewis et al. 2014).

### The antibacterial activity of the Au@AgNPs

Due to the emerging antibiotic resistance, several approaches are studied as alternatives to antibiotics. Among the suggested alternatives are the nanoparticles that can be administrated alone or in combination with antibiotics (Yallappa et al. 2015). Several studies have reported the antibacterial activity of AgNPs, and AuNPs (Kuppusamy et al. 2017; Beldjilali et al. 2020; Herbin et al. 2022). Moreover, recent studies supported that AgNPs have higher activity than AuNPs (Liu et al. 2016; Aldayel et al. 2022; Beck et al. 2022), which can be explained by the different functional groups attributed to both nanoparticles upon the biosynthesis (Li et al. 2018). Moreover, the alloy of the bi-metallic Au-AgNPs shows various promising applications in different fields, as many studies reported its higher reactivity compared to each of the nanoparticles (Emam et al. 2017; Fang et al. 2019; Ha Pham et al. 2021). Thus, the study investigates the possible antibacterial activity of the alloy Au@AgNPs using propolis extract.

In a study by Loo et al. they conducted disc diffusion, MIC and MBC, and time-killing curves of green synthesized AgNPs against Gram-negative bacteria, including *Escherichia coli *(*E. coli*), *Klebsiella pneumoniae*, *S.* Typhimurium, and *Salmonella* Enteritidis (Loo et al. 2018). Although Singh and Mijakovic biosynthesized silver and gold nanoparticles, they tested the antibacterial activity of biosynthesized silver nanoparticles only against two Gram-negative bacteria, including *P. aeruginosa* and *E. coli* (Singh and Mijakovic 2022). Moreover, Khan et al. biosynthesized silver and gold nanoparticles. They demonstrated their antibacterial activity separately against two Gram-positive bacterial strains (*Bacillus subtilis* and *Staphylococcus aureus*) and two Gram-negative bacterial strains (*Klebsiella* and *E. coli*) using the disc diffusion method and MIC (Khan et al. 2020).

Therefore, in this study, the antibacterial activity of the biosynthesized Au@AgNPs was tested against both Gram-positive antibiotic-resistant *S. sciuri*, and Gram-negative *P. aeruginosa*, and *S. enterica* by disc diffusion tests. Moreover, the MIC and MBC were determined. In addition, to understand more about the bacterial dynamics in response to the treatment with different concentrations of Au@AgNPs, different Gram-positive and Gram-negative bacteria were treated with the Au@AgNPs, and their growth was monitored over time. Then, the treated bacteria were visualized using SEM to study the effect of Au@AgNPs on the bacterial membrane integrity.

The results confirmed the antibacterial activity of the formulated Au@AgNPs, as indicated by the MIC and MBC results and the disc diffusion test. The results agreed with the previous studies on *Salmonella* (Duffy et al. 2018; Chen et al. 2019; Farouk et al. 2020), *P. aeruginosa* (Lara et al. 2010; Yan et al. 2018) and *Staphylococcus* bacteria (Elbehiry et al. 2019; Parvekar et al. 2020), where each of the nanoparticles were effective against the model bacteria in various antibacterial and clinical applications. Moreover, the bacterial dynamics upon treatment with Au@AgNPs indicate that the nanoparticles work in a concentration-dependent manner. Furthermore, the SEM micrographs corresponding to the heatmap showed signs of cell membrane damage which might be the reason behind the antibacterial effect of the formulated Au@AgNPs. This observation agreed with previous studies that reported cell membrane damage with various types of nanoparticles (Nisar et al. 2019; Vazquez-Muñoz et al. 2019; Abdelsattar et al. 2022). In addition to the cell membrane damage, nanoparticles lead to protein and DNA misfolding and reactive oxygen species (ROS) production were all reported as various mechanisms of antibacterial properties of the nanoparticle (Herbin et al. 2022). Further tests can be done to investigate the other action pathways of the formulated Au@AgNPs.

### Cytotoxicity and potential anticancer activity of Au@AgNPs

Gold and silver nanoparticles have various properties, making them involved in various medical approaches, including antibacterial and anticancer applications(Chugh et al. 2018). In a study by Lomelí-Marroquín et al. they showed that the biosynthesized Ag-Au nanoparticles could inhibit the cell proliferation of human melanoma cells (Lomelí-Marroquín et al. 2019). In addition, Shkryl et al. showed that the green synthesized Ag-Au nanoparticles exhibited cytotoxicity against mouse neuroblastoma and embryonic fibroblast cells, but the anticancer activity was not assessed (Shkryl et al. 2021). Accordingly, we intended to study the anticancer activity and cytotoxicity of our green synthesized Au@AgNPs on hepatocellular carcinoma cell lines. Various concentrations (from 10 to 1000 µg/mL) of the prepared Au@AgNPs were tested and compared to the untreated cell lines. These results indicated that the biosynthesized Au@AgNPs at low concentrations (including the concentration used for the antibacterial activity, 50 µg/mL) have a minimal effect on cell viability, which agreed with the previously reported results. Kamil Brzóska and his colleague measured the cell viability of HEPG2 with a selected concentration (10 µg/mL) of both AuNPs and AgNPs with no significant impact on the viability (Brzóska et al. 2019). However, at high concentrations (from 500 µg/mL and higher), the biosynthesized Au@AgNPs have a cytotoxic effect on HEPG2 cancer cell lines, with IC50 of 140 µg/mL. Earlier, AuNPs could inhibit the growth of around 97% of HEPG2 cells when using 250 µg/mL concentration (Muthukumar et al. 2016). Other work reported the cytotoxicity of AgNP with IC50 for both PLLAgNP and CTABAgNP to be around 25 mg Ag/L (Brkić Ahmed et al. 2017). Accordingly, our results have proven to be a promising formula for cancer treatment upon using lower concentrations than the reported in the literature of green synthesized bimetallic nanoparticles, especially Ag-Au nanoparticles. This finding suggested the potential anticancer activity of formulated Au@AgNPs.

### Conclusion

In conclusion, gold-silver nanoparticles (Au@AgNPs) were green synthesized using propolis extract by a novel and simple method. Moreover, they were evaluated as a safe and effective antimicrobial agent against both Gram-positive antibiotic-resistant *S. sciuri,* Gram-negative *P. aeruginosa*, and *S. enterica*. The biosynthesized nanoparticles were characterized using UV–visible spectrum, TEM, SEM, Zeta potential, FTIR, DLS and EDX. In addition, the antibacterial activity was assessed through disc diffusion assay, MIC, MBC, growth kinetics assay, and cell membrane integrity assay. Besides, the anticancer activity was assessed on hepatocellular carcinoma cell lines. The experiments exhibited that biosynthesized Au@AgNPs showed significant antibacterial activity against different Gram-positive and Gram-negative bacterial strains with a variable range of activity. In addition, the potential anticancer activity of the formulated nanoparticles when used in a concentration of 500 µg/mL or higher. As a result, this research revealed that green synthesized Au@AgNPs, which lead to membrane damage, could be effective anticancer and antibacterial agents against *S. sciuri*, *P. aeruginosa*, and *S. enterica*, with minimum cytotoxic effect at the working antibacterial concentration.

## Data Availability

All data are available.
